# Pandemic Perspective: Commonalities Between COVID-19 and Cardio-Oncology

**DOI:** 10.3389/fcvm.2020.568720

**Published:** 2020-12-04

**Authors:** Sherry-Ann Brown, Svetlana Zaharova, Peter Mason, Jonathan Thompson, Bicky Thapa, David Ishizawar, Erin Wilkes, Gulrayz Ahmed, Jason Rubenstein, Joyce Sanchez, David Joyce, Balaraman Kalyanaraman, Michael Widlansky

**Affiliations:** ^1^Cardio-Oncology Program, Division of Cardiovascular Medicine, Medical College of Wisconsin, Milwaukee, WI, United States; ^2^Division of Cardiovascular Medicine, Medical College of Wisconsin, Milwaukee, WI, United States; ^3^Division of Hematology and Oncology, Medical College of Wisconsin, Milwaukee, WI, United States; ^4^Department of Pharmacy, Froedtert Health and Medical College of Wisconsin, Milwaukee, WI, United States; ^5^Division of Infectious Diseases, Medical College of Wisconsin, Milwaukee, WI, United States; ^6^Division of Cardiothoracic Surgery, Medical College of Wisconsin, Milwaukee, WI, United States; ^7^Department of Biophysics, Medical College of Wisconsin, Milwaukee, WI, United States

**Keywords:** cardio-oncology, COVID-19, pandemic, telemedicine, inflammation, cytokine release syndrome, right ventricle, health disparities

## Abstract

Overlapping commonalities between coronavirus disease of 2019 (COVID-19) and cardio-oncology regarding cardiovascular toxicities (CVT), pathophysiology, and pharmacology are special topics emerging during the pandemic. In this perspective, we consider an array of CVT common to both COVID-19 and cardio-oncology, including cardiomyopathy, ischemia, conduction abnormalities, myopericarditis, and right ventricular (RV) failure. We also emphasize the higher risk of severe COVID-19 illness in patients with cardiovascular disease (CVD) or its risk factors or cancer. We explore commonalities in the underlying pathophysiology observed in COVID-19 and cardio-oncology, including inflammation, cytokine release, the renin-angiotensin-aldosterone-system, coagulopathy, microthrombosis, and endothelial dysfunction. In addition, we examine common pharmacologic management strategies that have been elucidated for CVT from COVID-19 and various cancer therapies. The use of corticosteroids, as well as antibodies and inhibitors of various molecules mediating inflammation and cytokine release syndrome, are discussed. The impact of angiotensin converting enzyme inhibitors (ACEIs) and angiotensin receptor blockers (ARBs) is also addressed, since these drugs are used in cardio-oncology and have received considerable attention during the COVID-19 pandemic, since the culprit virus enters human cells *via* the angiotensin converting enzyme 2 (ACE2) receptor. There are therefore several areas of overlap, similarity, and interaction in the toxicity, pathophysiology, and pharmacology profiles in COVID-19 and cardio-oncology syndromes. Learning more about either will likely provide some level of insight into both. We discuss each of these topics in this viewpoint, as well as what we foresee as evolving future directions to consider in cardio-oncology during the pandemic and beyond. Finally, we highlight commonalities in health disparities in COVID-19 and cardio-oncology and encourage continued development and implementation of innovative solutions to improve equity in health and healing.

## Introduction

In early 2020, the World Health Organization (WHO) designated the new, highly contagious, and unnervingly fatal disease COVID-19 caused by the novel severe acute respiratory syndrome coronavirus 2 (SARS-CoV-2) a global pandemic. By June 1, 2020, the WHO reported more than 6 million confirmed cases and 370,000 deaths across nearly 220 countries and territories, with the US having the highest number of confirmed cases (1.7 million) and deaths (100,000) (1).

Although initially thought to be primarily a lung disease, COVID-19 also involves marked toxicity to the cardiovascular system. As data has emerged, it has become clear to our cardio-oncology group (2–7) that much of the cardiovascular toxicity reported in COVID-19 is also observed in cardio-oncology, with overlap in underlying pathophysiology. Additionally, pharmacologic options frequently used or currently being studied in cardio-oncology are also proving beneficial in COVID-19. This begs the question of whether evaluating commonalities in the toxicities, pathophysiology, and pharmacology of COVID-19 and cardio-oncology would be informative for advancing understanding and avenues for research in cardio-oncology, as well as COVID-19. Cardio-oncology is an emerging field in medicine focused on the prevention, surveillance, detection, and management of injury to the cardiovascular system from cancer therapies or from cancer itself. The cardiovascular injuries are inflicted by an exogenous source, primarily pharmacologic or radiologic cancer therapy. In COVID-19, the cardiovascular injuries are also incited by an exogenous source, primarily SARS-CoV-2. Due to the exogenous nature of the original source of injury, in addition to pathophysiology mediating the injury, some authors refer to these cardiovascular injuries in COVID-19 as “toxicities” (8–10), which is also the term conventionally used in cardio-oncology (11–13). While cancer therapies and SARS-CoV-2 are two very different entities, the havoc they both wreak on the cardiovascular system is thought-provoking.

In this perspective, we share the overarching viewpoint that these commonalities exist and are intriguing, and consequently, the dynamic research efforts surrounding COVID-19 may be able to inform new understanding and avenues for investigation in cardio-oncology. A clear understanding of the mechanisms of various forms of CVT in cardio-oncology remains elusive. Development of novel concepts, paradigms, and drug utilization trends based on observations identified in CVT related to COVID-19 may help advance research and clinical practice in cardio-oncology. To this end, we first present cardiovascular toxicities common to COVID-19 and cardio-oncology, then we expound on underlying pathophysiology. This is followed by description of pharmacologic options being pursued in both COVID-19 and cardio-oncology. Finally, we discuss ramifications of these commonalities in the context of Cardio-Oncologic care and research in the pandemic and beyond (Table 1).

**Table 1:** Mechanisms, concepts, and paradigms: commonalities in toxicity, pathophysiology, and pharmacology of cardio-oncology and COVID-19.

**Common topic of study**	**Cardio-oncology**	**COVID-19**
Mechanisms of left ventricular cardiomyopathy	Elucidate mechanisms and optimal management of left ventricular systolic dysfunction in Cardio-Oncology	Elucidate mechanisms and optimal management of left ventricular systolic dysfunction in COVID-19
Immune system activation	Analyze pathophysiology and optimal management of immune response, cytokine release syndrome, and autoimmune adverse effects from ICIs or CAR-T cell therapy	Analyze pathophysiology and optimal management of immune response, cytokine release syndrome, and related adverse effects in COVID-19
Long-term sequelae of inflammation	Investigate long-term implications of inflammation induced by neoplastic agents	Investigate long-term implications of myocardial inflammation in COVID-19
Endothelial dysfunction	Interrogate role of endothelial dysfunction in ischemic and cardiomyopathic cardiovascular injuries from cancer drugs	Interrogate role of endothelial dysfunction in ischemic and cardiomyopathic cardiovascular injuries from COVID-19
Coagulopathy and anticoagulation	Study the burden, mechanisms, and optimal management of coagulopathy (arterial or venous) with need for anticoagulation or antiplatelet therapy in Cardio-Oncology	Study the burden, mechanisms, and optimal management of coagulopathy and microthrombosis with beneficial response to anticoagulation in COVID-19
Role of RV and RVAD	Explore significance of RV systolic dysfunction after anthracycline therapy	Explore significance of RV systolic dysfunction in severe COVID-19 infection
Prognostic value of RV strain	Evaluate utility of RV strain to predict outcomes following anthracycline therapy	Evaluate utility of RV strain to predict COVID-19 severity/mortality
Utility of steroid therapy and biologics	Determine the effectiveness and timing of steroid treatment and monoclonal antibodies for inflammation- or immune-related adverse events from ICIs or CAR-T cells	Determine the effectiveness and timing of steroid treatment and monoclonal antibodies for inflammation-related adverse CV events in COVID-19
Neurohormonal therapy	Establish cardioprotective contributions of neurohormonal therapies	Establish whether neurohormonal therapies are protective in COVID-19
Potential drug Interactions	Appraise the extent and impact of potential drug interactions between Cardiology drugs and Oncology drugs	Appraise the extent and impact of potential drug interactions between Cardiology drugs and COVID-19 drugs
Impact of health disparities	Assess underlying factors and solutions to address health disparities in cardiovascular toxicities observed in Cardio-Oncology	Assess underlying factors and solutions to address health disparities observed in cardiovascular injuries in COVID-19
Precision of risk prediction	Develop precise methods of predicting cardiovascular toxicities and prognosis	Develop precise methods of predicting risk and overall prognosis in COVID-19

*CAR-T Cells, Chimeric Antigen Receptor T-Cells; COVID-19, Coronavirus Diseases of 2019; CV, cardiovascular; ICI, Immune Checkpoint Inhibitor; RV, Right Ventricle; RVAD, Right Ventricular Assist Device*.

## Common Toxicities in COVID-19 and Cardio-Oncology

### CVT in COVID-19 and Cardio-Oncology

In COVID-19, SAR-CoV-2 causes direct and indirect cardiovascular injury, which typically manifests as cardiomyopathy, myopericarditis, ischemia, or arrythmia (14–25). SARS-CoV-2 has been discovered in cardiac tissue (15, 25), similar to SARS-CoV-1 infection in which 35% of patients had viral RNA expressed in cardiac tissue (26). Patients with pre-existing CVD and cancer or CVD risk factors (e.g., diabetes mellitus, chronic kidney disease, obesity, and advanced age) are among those at highest risk of poor outcomes, i.e., increased morbidity and mortality from COVID-19 (10, 22, 27–30). According to a retrospective analysis of 72,314 cases in China, patients with pre-existing CVD morbidities had a five-fold increase in mortality, and a COVID-19-related death rate of 10.5% (22). Indirectly, patients with CVD morbidities are inherently more susceptible to the adverse effects of viral infection and the body's adaptive response. The systemic effects of COVID-19 causing fever, hypoxia, hypotension, and tachycardia may not be well-tolerated in patients with underlying cardiomyopathy or obstructive coronary artery disease, and this may manifest as further myocardial injury, and increased incidence of decompensated heart failure and type II myocardial infarction (20). Evidence of myocardial injury (e.g., elevated troponin), is common in patients hospitalized with COVID-19 (10). When present, elevated cardiac biomarkers such as brain natriuretic peptide and serum troponin have been associated with increased mortality in patients with COVID-19 (18).

Similarly, a wide spectrum of cancer therapies has been associated with CVT, such as cardiomyopathy, myopericarditis, ischemia, and arrhythmias (11, 31). Radiation therapy can lead to all of these toxicities in the absence of chemotherapy. Various chemotherapy and targeted cancer therapy regimens can also result in CVT. Anthracyclines most commonly associate with cardiomyopathy, and can also bring about conduction abnormalities, myocarditis, or pericardial disease. Tyrosine kinase inhibitors commonly associate with hypertension, and less commonly with cardiomyopathy or ischemia. Immune checkpoint inhibitors are most notorious for myocarditis, and can also prompt pericarditis, cardiomyopathy, conduction abnormalities, and ischemia. Many other CVT are noted in cardio-oncology, with a variety of drug classes. In addition, tachycardia and elevated biomarkers may also portend poor prognosis in cardio-oncology (32, 33). There is therefore much overlap of CVT and prognostic factors in cardio-oncology with CVT and prognostic biomarkers in COVID-19. Furthermore, some types of cancers and cancer treatments weaken patients' immune systems and increase risk of any infection. Cancer patients' immunosuppression often also associates with blunted or delayed symptoms, which could in turn delay urgent therapy and increase mortality in COVID-19.

Interesting to consider is any potential synergistic CVT in patients on cancer therapies in COVID-19. A prospective cohort of 800 cancer patients with COVID-19 analyzed in late April 2020 linked COVID-19 mortality with older age, male gender and comorbidities such as hypertension and cardiovascular disease (14). There was no association between receipt of cytotoxic chemotherapy, targeted therapies, radiation therapy, or other cancer therapies and COVID-19 mortality in this cohort. Similarly, a retrospective cohort of 928 cancer patients from the USA, Canada and Spain associated advanced age, smoking, progressive malignancy and increased comorbidities COVID-19 mortality, but failed to show associations with cancer type and type of anticancer therapy with COVID-19 mortality (34).Thus, the role of chemotherapy and other cancer systemic therapies in COVID-19 mortality remains uncertain.

### Emerging Role of Right Ventricular Failure

Recent studies emerging in parallel in the pandemic and in cardio-oncology indicate that the RV may play an important role in the prognosis of patients with COVID-19 or CVT from cancer therapy; RV failure generally associates with worse outcomes in a variety of populations, and patients with COVID-19 or CVT from cancer therapies may be no different (35–38). While the mechanisms of insult to the RV in COVID-19 are different from those in cardio-oncology, similar changes are noted in the ventricle and these may have prognostic value. Importantly, RV longitudinal strain (RVLS) has emerged as a key player in the prediction of RV failure in both COVID-19 and cardio-oncology (36, 38).

In COVID-19, RVLS inversely associates with myocardial injury, mechanical ventilation, acute respiratory distress syndrome (ARDS), and mortality, as well as signs of systemic inflammation such as heart rate, D-dimer, and C-reactive protein, as well as thromboembolism (38). In addition to RVLS, RV dilation and systolic dysfunction also predict mortality in COVID19 (39). Abnormalities in RV strain, size, and systolic function in COVID-19 may result from ARDS, pulmonary hypertension with increased pulmonary vascular resistance due to acute lung injury or thromboembolism, in addition to CO_2_ retention, positive pressure ventilation, or other causes of acute myocardial injury (19, 40–46). In one COVID-19 study, of 10 patients with RV dilation, 50% had PE noted on CTA; and of 21 total deaths in that COVID-19 cohort, 62% had RV dilation (39). Some patients with apparent ARDS do not respond as expected to low pressure ventilation strategies per ARDSNet ventilation protocols (47). Prone positioning in COVID-19 improves oxygenation and reduces the risk and need for mechanical ventilation or extracorporeal membrane oxygenation (ECMO) in patients on mechanical ventilation, but the maneuver appears to also reduce the risk of RV failure in ARDS including COVID-19 (48–50). Anecdotally, we have observed that the typical progression from hypoxemic respiratory failure to multi-system organ failure with escalating pressor requirements can be blunted with insertion of a percutaneous right ventricular assist device (RVAD) connected to an oxygenator. In all cases, the pressor requirement has been eliminated upon initiation of RVAD flows. These observations are consistent with our experiences in using these devices to treat other forms of RV failure which are frequently misdiagnosed as distributive shock.

In cardio-oncology, changes are also noted in RV strain, structure, function, and size in patients with breast cancer and lymphoma who receive anthracycline chemotherapy (36, 51). Although the left ventricle is more commonly studied, the RV also shows impairment in contractility, with temporal changes of decreased RVLS and increased right ventricular end systolic volume (RVESV) preceding reduction in right ventricular ejection fraction (RVEF) (36). Additionally, patients with end-stage heart failure as a result of cardiomyopathy from anthracycline therapy benefit from RV assist device support (52). The underlying pathophysiology of RV dysfunction in anthracycline CVT is likely similar to LV dysfunction. LV dysfunction results from release of cytokines and inflammatory markers, related to generation of reactive oxygen species, disruption of mitochondrial biogenesis, and activation of apoptosis, and double-stranded DNA breaks (53–55). This is a recent and novel area of inquiry in cardio-oncology. Additional studies are needed to determine whether RV size, function, and longitudinal strain can predict CVT and mortality in cardio-oncology (36), as has been found in COVID-19.

### Health Disparities in CVT

A multi-ethnic study of more than 3,500 individuals with COVID-19 was published in the New England Journal of Medicine (NEJM) (56). While <40% of patients in the study were hospitalized, African Americans composed almost 80% of inpatients admitted with COVID-19 and associated CVT. A higher rate of comorbidities associated with the risk for hospitalization, and African Americans had higher rates of comorbidities. This is similar to general trends in health disparities, in which African Americans have higher rates of CVD, obesity, hypertension, and diabetes than Caucasians (57), and are therefore at higher risk for CVT related to COVID-19. These disparities were found to associate with inequities in socioeconomic demographics in the NEJM report, as in prior studies (56, 57). Notably, ACE (I/D) polymorphisms have been implicated in COVID-19 and related CVT, and vary across racial groups (58). However, this alone does not explain the disparities observed in COVID-19. The D/D polymorphism that associates with the development and severity of sarcoidosis (59), which is more prevalent, complex, and mortal in African-Americans (60), is the same polymorphism that is suggested to associate with protection in COVID-19 (61–63). Nevertheless, African Americans have had the highest proportions of severe and fatal illness from COVID-19 and consequent CVT. In the same way, CVT in cardio-oncology has been reported at higher rates in African Americans, with similar underlying reasons (64–68).

### Implications of Common Toxicities

**Figure 1:**
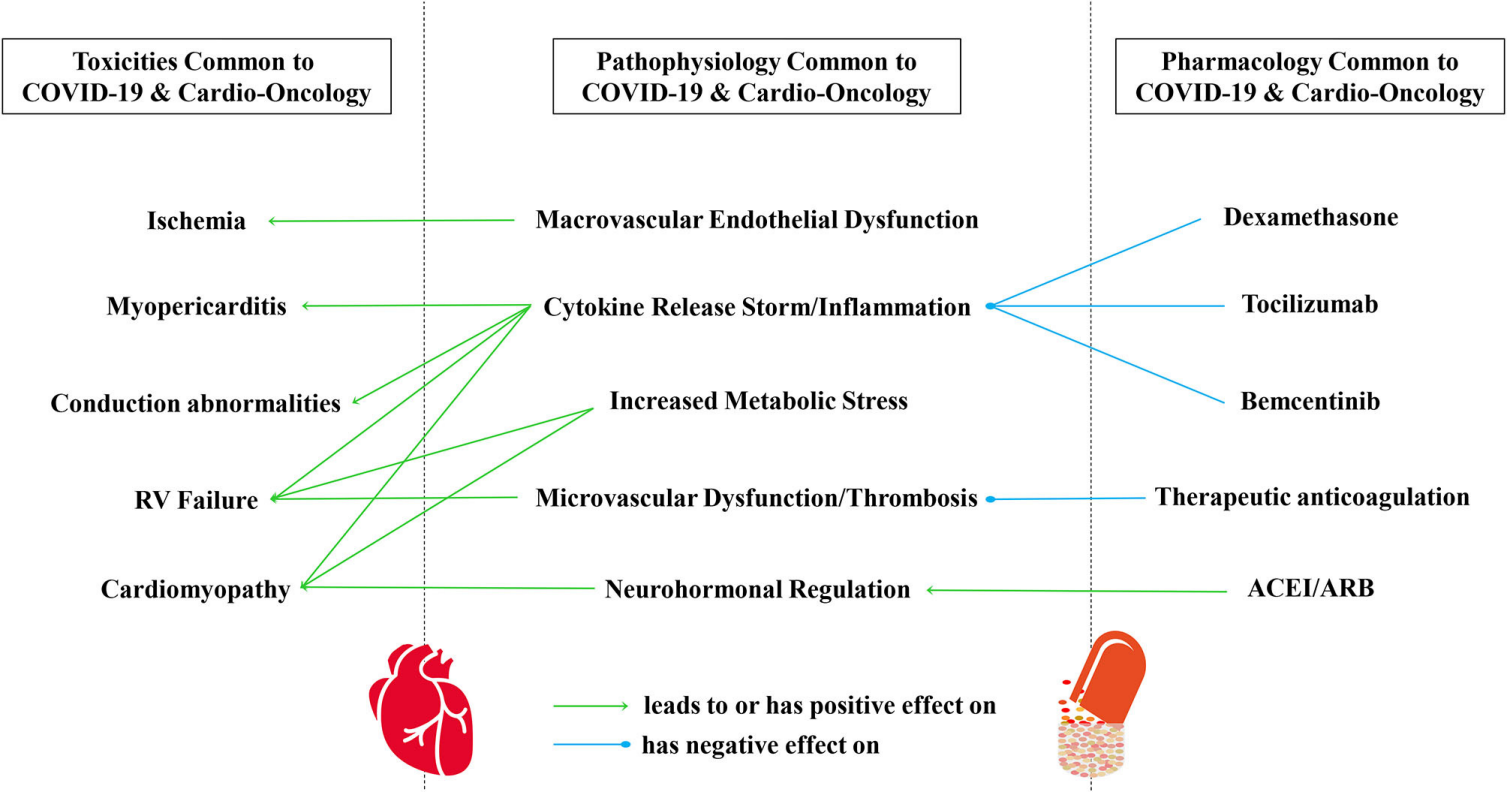
A conceptual framework of commonalities in CV toxicities, pathophysiology, and pharmacology common to COVID-19 and cardio-oncology. CV toxicities common to COVID-19 and cardio-oncology include myocardial injury, cardiomyopathy, myopericarditis, ischemia, conduction abnormalities, and RV failure, in part mediated by immune system activation, cytokine release syndrome, and arterial and venous coagulopathy. All of these are also examples of oncologic CV toxicities that can result from pharmacologic or radiation therapies. Indeed, the pathophysiology and modulation of SARS-CoV-2 infection remains under investigation, with components of viral infection/invasion, macrovascular endothelial dysfunction, cytokine release syndrome/inflammation, microvascular dysfunction/thrombosis, neurohormonal regulation, coagulopathy, and increased metabolic stress. Several pharmacologic considerations have risen to the surface during the pandemic, involving steroids, cancer immunotherapy, biologic antibodies and inhibitors, drug repurposing, the role of cyp450 and drug transporters in drug-drug interactions, anticoagulation, and neurohormonal regulation. ARB, Angiotensin Receptor Blocker; ACEI, Angiotensin Converting Enzyme Inhibitor; CV, Cardiovascular; COVID-19, Coronavirus Disease of 2019; RV, Right Ventricle; SARS-CoV-2, Severe Acute Respiratory Syndrome Coronavirus.

It is worth continuing to study shared toxicities in COVID-19 and cardio-oncology. For example, increased attention to emerging special topics such as RV strain, function, and predictive value in COVID-19 may help elucidate sequelae of commonalities to optimize care and survival of our patients in COVID-19 and also in cardio-oncology (Figure 1). Perhaps studying the pathophysiology and host characteristics in patients with abnormal RV size, function, and longitudinal strain in COVID-19 could also help us better understand the pathophysiology of abnormal RV size, function, and longitudinal strain in some patients after anthracycline therapy. Further, it will be important to address the disproportionate percentages of African-Americans with severe and fatal CVT related to both COVID-19 (69–73) and cancer therapies in cardio-oncology (64–68).

## Common Pathophysiology in COVID-19 and Cardio-Oncology

### Pathophysiology of COVID-19 and Cardio-Oncology

Potential mechanisms of cardiovascular injury in COVID-19 include hemodynamic derangement or hypoxemia, increased metabolic stress, demand ischemia, microvascular dysfunction or thrombosis due to hypercoagulability, or systemic inflammation and cytokine storm, which may also destabilize existing coronary artery plaques (16, 74–76). Although not yet demonstrated with SARS-CoV-2, an autopsy study of people who died from SARS-CoV-1 infection demonstrated that 35% of patients had viral RNA expressed in cardiac tissue (26). Further, a recent study illustrated *in vitro* direct infection of human induced pluripotent stem cell-derived cardiomyocytes (hiPSC-CMs) by SARS-CoV-2 (77). Microscopy and RNA-sequencing provided evidence that SARS-CoV-2 enters hiPSC-CMs *via* the cell surface receptor ACE2. The study also demonstrated that in response to SARS-CoV-2 infection, the hiPSC-CMs upregulated the innate immune response and antiviral clearance gene pathways, in addition to downregulating ACE2 expression.

ACE2 receptors are the SARS-CoV-2 entry point into human cells (10, 78). Patients with pre-existing CVD or CV risk factors, which associate with heightened systemic inflammation, have higher levels of ACE2 receptor expression than the general population (10, 79, 80). In normal physiology, ACE2 is counter-regulatory and anti-inflammatory (79, 80). Interestingly, a particular angiotensin converting enzyme (ACE) genetic polymorphism (D/D), although not a ACE2 polymorphism, associates with decreased ACE2 levels and has been suggested to be protective in patients with COVID-19 (61–63). The physiologic effects of ACE and ACE2 are typically in some degree of homeostatic equilibrium, with ACE mediating inflammation, oxidative stress, and vasoconstriction, and ACE2 also being vasodilatory (81). SARS-CoV-2 may remove ACE2 from this homeostatic pathway due to both the virus and the receptor being internalized from the cell surface in COVID-19 (81).

The inflammatory response elicited by SARS-CoV-2 is implicated in direct suppression of cardiac contractility (75). Evidence of new contractile dysfunction was reported in ~30% of patients with critical illness related to COVID-19, and cardiac or circulatory shock is a common pathway to fatal outcomes (82, 83). This is reminiscent of CVT in cardio-oncology, in which increased metabolic stress, cytokine release, inflammation, macrovascular endothelial dysfunction, microvascular dysfunction, thrombosis, and neurohormonal dysregulation can all result in impairment of cardiac contractility underlying cardiomyopathy.

### Immune System Activation

Two recent studies evaluating immunologic characteristics of peripheral blood samples from COVID-19 patients have emerged from China (84, 85). In these studies, severe cases of COVID-19 were associated with depletion of CD8+ T-cells, suggesting that upregulation of immune checkpoint molecules that downregulate T-cells may play an important role in impairing the immune response to the virus. These early studies should be interpreted with caution given the small sample sizes, and continued investigation will shed light on the mechanisms of immune dysregulation induced by COVID-19.

Immune checkpoint inhibitors (ICIs) are drugs that target immune checkpoint molecules such as programmed death 1 (PD-1), programmed death-ligand 1 (PD-L1), and cytotoxic T-lymphocyte-associated protein 4 (CTLA-4). These drugs have dramatically improved overall survival for patients with a wide range of malignancies (86). Inflammatory cytokines, such as interferon-γ and type I interferons, induce PD-L1 expression on immune and tumor cells (87). Interaction of the PD-L1 and PD-1 proteins leads to T-cell exhaustion, and blockade of this interaction with PD-1/PD-L1 inhibitors restores effector function to CD8+ T-cells, allowing for destruction of malignant cells. Chief among concerns with ICIs during the pandemic is whether ICIs can increase COVID-19-related complications, particularly CVT. A retrospective study found patients receiving ICIs to be at higher risk of hospitalization and severe outcomes from COVID-19 (88). Strong conclusions are difficult to draw from this small, retrospective, single-center study in which only 31 patients received ICIs. A prospective observational study from the UK Coronavirus Center Monitoring Project found no association between COVID-19 mortality and ICI treatment in the 44 patients who received ICIs (89). Ongoing large-scale prospective data may shed further light on this interaction.

Many cancer patients receiving ICIs possess comorbidities that enhance risk for poor outcomes related to COVID-19. ICIs and COVID-19 can cause overlapping organ toxicities, particularly pulmonary and cardiac, which inform risk-benefit decisions on ICI use during the pandemic. ICIs can induce immune-mediated cardiotoxicity, including myocarditis, pericarditis, heart failure, arrhythmias, and MI. These events are uncommon, occurring in <3% of patients who receive ICIs, but carry high risk of mortality (90).

The pathophysiology of the immunologic mechanisms of cardiotoxicity with ICIs and COVID-19 likely differ, but macrophages may play roles in both pathways, which could contribute to anecdotal response to glucocorticoid responsiveness for ICI and COVID-19 toxicities (Table 2). The renin-angiotensin system has been implicated in the pathophysiology of both COVID-19 and tumorigenesis, with data suggesting the RAAS pathway promotes an immunosuppressive tumor microenvironment (91–93). However, much like COVID-19, the impact of ACE inhibitors on survival outcomes with ICIs is currently unclear (94). Given the prevalence of ICI use, it is essential to exert a coordinated effort to track COVID-19 incidence in patients receiving ICIs, as well as rates of pulmonary and cardiac sequelae and mortality to truly understand the long-term impact of the virus on this large population.

**Table 2:** Clinical characteristics of similar CV toxicity in ICI therapy, CAR T-cell therapy, and COVID-19.

	**ICI and CV toxicity**	**CAR-T cell and CV toxicity**	**COVID-19 and CV toxicity**
Incidence	<1%	NA/unknown	NA/unknown
Pathophysiology	T-cell mediated	Cytokine storm, high IL-6	Hypoxia, Cytokine storm
Risk of VTE	High	High	High
Management	Steroid	Tocilizumab, and/or steroid	Supportive management, +/– dexamethasone
ACEI/ARB continue	Yes	Yes	Yes
Long-term CV effect	Unknown	Unknown	Unknown

*ACEI, ACE Inhibitor; ARB, Angiotensin Receptor Blocker; CAR-T Cells, Chimeric Antigen Receptor T-Cells; CRS, Cytokine Release Syndrome; CV, Cardiovascular; COVID-19, Coronavirus Diseases of 2019; ICI, Immune Checkpoint Inhibitor; IL-6, Interleukin 6; NA, Not Applicable; VTE, Venous Thromboembolism*.

### Cytokine Release Syndrome

In COVID-19, the inflammatory cytokine IL-6 has also been shown to play a role in critically ill patients, in whom “cytokine release storm” or “cytokine release syndrome” (CRS) pathophysiology leads to cardiopulmonary complications and multisystem failure (95). Clinical manifestations of CRS include fever, chills, fatigue, myalgias, arthralgias, nausea, vomiting, and diarrhea (96). In the patient with CRS, cardiovascular manifestations include tachycardia, hypotension, elevated troponin, heart failure, and in severe cases, cardiogenic shock (96, 97). IL-6 could possibly mediate cardiac dysfunction and hemodynamic instability (98). In general, IL-6 elevation has associated with cardiovascular complications such as atherosclerosis, MI, and heart failure.

IL-6 and other cytokines are key components of the human body host defense system against infection, yet high levels of these cytokines in a hyperinflammatory response can lead to CRS (99, 100). Cytokine release syndrome can be a fatal complication due to exaggerated inflammatory response in COVID-19, partially mediated by immune cells fighting the viral infection by increasing inflammatory cytokines *via* activation of intracellular NF-κB (101), but also in large part mediated by the ACE2 and AT1 receptors, which are generally highly expressed on epithelial cells in the lung and endothelium (20, 102–104). A main function of ACE2 is to convert angiotensin II (Ang II) into angiotensin-(1-7), a counter-regulatory peptide that dampens the inflammatory effects of Ang II *via* AT1 (101, 105). After the SARS-CoV-2 S-protein attaches to ACE2 on respiratory epithelium, ACE2 is down-regulated (77). The resulting SARS-CoV-2-mediated imbalance of serum Ang II/angiotensin-(1-7) drives net activation of AT1 signaling [which is dependent on serum Ang II/angiotensin-(1-7)] in pulmonary epithelial cells. It is well-established that COVID-19 infection causes hyperactivation of the angiotensin 1 receptor (AT1), which leads to disproportionate activation of nucleotide-binding domain-like receptor protein 3 (NLRP3) inflammasome in lung epithelial cells and endothelium (106, 107), as well as activation of STAT3 and the NF-κB pathway, producing potent pro-inflammatory cytokines IL-6, IL-1β, and IL-18 (101, 108–110). The detrimental pathophysiological consequence of the hyperinflammatory response includes enhanced activation of reactive oxygen species (ROS) release, fibrosis, vasoconstriction, and programmed cell death, that contribute to the CRS pathophysiology. Interestingly, ACE2 and AT1 are known to be expressed at extremely low levels on hematopoietic stem cells (HSC) and endothelial progenitor cells (EPC) (111). A recent study demonstrated for the first time that ACE2 is expressed on very small embryonic-like stem cells (VSELs) (112). Pre-clinical data demonstrated that interaction of ACE2 receptor with the COVID-19 spike protein activated the NLRP3 inflammasome in VSELs and HSC leading to programmed cell death (112); the contribution of this to CRS is yet unclear.

Unlike traditional chemotherapy, CAR-T cell therapy is a novel form of immunotherapy in cardio-oncology to treat individuals with refractory hematologic malignancies, and is commonly associated with toxicity related to CRS. CAR-T cell therapy utilizes genetically engineered T-cells to attack cancer cells (113). The activation of CAR-T cells when engaged with antigen in a malignant cell leads to its CAR-T cell proliferation, which further activates monocytes and macrophages, leading to release of proinflammatory cytokines and chemokines such as IL-6, IL-8, IL-10, interferon-gamma (INF-y), monocyte chemoattractant protein-1b, and granulocyte-macrophage colony-stimulating factor (114, 115). These proinflammatory cytokines are potential mediators for CRS in patients with cancer, with a similar cascade in patients with COVID-19.

### Coagulopathy

Arterial and venous coagulopathy has emerged as an important factor in COVID-19 pathophysiology and cardio-oncology, especially in critically ill patients (116–120), in part related to underlying endothelial cell dysfunction and inflammation in patients with COVID-19 or cancer (117, 121, 122).

Severe COVID-19 infection requiring critical care admission has been associated with increased incidence of venous thromboembolism (VTE) (117, 123), due to hyperinflammation and a hypercoagulable state (124, 125). The incidence has been reported to be 3 to 4-fold greater than in the general population (117, 123). In critically ill patients in the general population, the cumulative incidence of VTE is around 9.6% (126, 127), while in COVID-19 patients it is reported to be between 31 and 42% (117, 123). Thrombotic events in COVID-19 mostly categorize VTE, but in some patients, a significant number of arterial thrombosis are also being reported. In one study, 3.7% of the 31% reported cases had ischemic strokes, while in another study population two ischemic strokes and one limb ischemia were reported (117, 123). Endotheliitis with underlying hyperinflammation, along with hypoxia leading to increased blood viscosity, are suspected to cause increased coagulopathy in severe COVID-19 infection (128, 129). Excess cytokine release also results in macroscopic or microscopic endothelial injury, leading to a prothrombotic state (130). Elevation of D-dimer above normal values on admission or over time during the disease process has been associated with poor outcomes in patients with severe COVID-19 (125). Close monitoring of D-dimer, aPTT/PT, fibrinogen, and platelet count in hospitalized COVID-19 patients is recommended as derangement of these coagulation parameters can be an early sign of disseminated intravascular coagulation (DIC) (125).

A similar phenomenon is observed in cancer patients (131). Similar factors are associated with thrombosis, with circulating microparticles, procoagulants, and endothelial dysfunction contributing to disruption of normal blood flow and hyperviscosity (120, 132, 133). Cancer also poses a 4 times increased risk of VTE as compared to general population while chemotherapy increases the risk to 6.5 times (134). Patients who receive CAR-T cell therapy are also at increased risk for venous thromboembolism, potentially mediated by CRS and high levels of IL-6 (135, 136), in the setting of underlying hypercoagulability due to the presence of the cancer itself. Other pharmacologic cancer therapies can also associate with thrombosis. Cisplatin and tyrosine kinases often lead to coronary or peripheral arterial thrombosis related to endothelial injury, thromboxane synthesis, and platelet activation and aggregation, placing patients at 1.5- to 1.7-fold or as high as 6-fold increased risk of acute coronary syndromes [see review in expert consensus statement (137)].

### Endothelial Dysfunction

Furthermore, many chemotherapeutics associate with endothelial dysfunction and consequent ischemia in the absence of thrombosis. In these cases, ischemia is due to vasospasm. This phenomenon can be caused by 5-fluorouracil (5-FU), capecitabine (5-FU pro-drug), paclitaxel, docetaxel, cisplatin (especially when combined with bleomycin or vincristine), cyclophosphamide, and tyrosine kinase inhibitors (e.g., sorafenib and sunitinib) [see review in expert consensus statement (137)]. Undiagnosed underlying coronary artery disease is thought to be a likely pre-disposing condition. Likewise, endothelial dysfunction and consequent ischemia in the absence of thrombosis are also suspected in some patients with COVID-19 who present with ACS and non-obstructed coronary arteries; severe hypoxia, CRS, plaque rupture, vasospasm, and microthromboembolism are also on the differential in these patients (25, 138, 139).

### Implications of Common Pathophysiology

Shared pathophysiology in COVID-19 and cardio-oncology also have important implications. For example, ICIs and CAR-T cells used as cancer therapy can lead to excessive activation of the immune system and inflammation and subsequently autoimmune and inflammatory adverse CV effects. Despite the favorable responses from CAR-T cell and ICI therapy in cardio-oncology, we still have limited evidence and understanding of CVT from these immunotherapies or their long-term impact. We can potentially fill our knowledge gap on CVT due to CRS related to CAR-T cell therapy, or supranormal activation of the immune system related to ICIs, in cardio-oncology by pursuing a better understanding of the inflammatory pathophysiology from the ongoing COVID-19 pandemic. The reverse is also true, and long-term sequelae of CRS on the cardiovascular system should be investigated and addressed in patients who have had COVID-19 or in cancer patients who have received CAR-T cell therapy. Similarly, coagulopathy in COVID-19 is an emerging topic with the majority of evidence stemming from observational studies and autopsies (128). The hypercoagulability of cancer, which is often treated by cardio-oncologists, can be informative for COVID-19, given a role for anticoagulation to address thromboembolism in these hypercoagulable states. Additionally, the role of endothelial dysfunction can be further elucidated in both COVID-19 and cardio-oncology with anticipated shared vascular pathophysiology, albeit with different mechanisms of endothelial injury.

## Common Pharmacology in COVID-19 and Cardio-Oncology

### Corticosteroids

Given the robust inflammatory response induced by COVID-19, corticosteroids are under investigation and have been demonstrating promising efficacy for treating the disease. Dexamethasone has recently garnered significant international attention for the treatment of COVID-19 with the pre-print publication (not yet peer reviewed) of the phase 3 RECOVERY trial. Patients were randomized to dexamethasone at 6 mg daily for up to 10 days vs. standard care. Dexamethasone significantly reduced deaths in patients who required supplemental oxygen or mechanical ventilation (140). Notably, the pre-print manuscript does not quantify the number of patients with cancer included in the analysis and may be difficult to generalize to an oncology population with COVID-19.

Corticosteroids have been mainstays of treatment for immune-related adverse events (irAEs) induced by ICIs and CAR-T cells in cancer patients, owing to their ability to rapidly dampen inflammation and quickly reverse irAEs (141, 142). In the widely utilized, evidence-based irAE management guidelines published by the American Society of Clinical Oncology (ASCO), high-dose corticosteroids are recommended as first-line management of most grade 2 or higher irAEs (143). For cardiovascular irAEs, including myocarditis, pericarditis, heart failure, and vasculitis, high-dose corticosteroids are recommended for any grade of toxicity (141, 142, 144). Thus, steroids may be helpful to quell activated immune responses leading to CVT due to various endogenous sources, whether cancer therapy or COVID-19.

### Biologic Antibodies and Inhibitors

Tocilizumab is an IL-6 receptor antagonist and is indicated as the first-line agent for the management of CRS in cancer patients (145–148). The use of tocilizumab in COVID-19 is an extrapolation based on the evidence of promising outcomes from using the drug to treat CRS from CAR-T cell therapy in cancer patients. Off-label use of tocilizumab is an option used in the management of severe cases of COVID-19 on compassionate grounds, supported by a case series from China (149) and a pilot open, single-arm multicenter study from Italy (150), particularly if tocilizumab is administered within 6 days of admission (HR 2.2, 95% CI 1.3–6.7, *p* < 0.05) (150). Additionally, a large retrospective cohort study demonstrated that tocilizumab decreased risk of death or minimized risk for invasive mechanical ventilation in patients with severe COVID-19 (adjusted HR 0.61, 95% CI 0.40–0.92; *p* = 0.020) (151). A smaller, retrospective cohort study demonstrated a significantly shorter need for vasopressor support in severely ill COVID-19 patients who received tocilizumab (152).

Cardiac dysfunction due to CRS is largely reversible, and in severe cases mitigated by tocilizumab (153). In some severe cases not responding to tocilizumab, the corticosteroid is added. In rare cases, when the patient does not respond to tocilizumab or steroid, other agents such as anakinra (IL-1R inhibitor) and etanercept (anti-TNFα) are potential options to hinder inflammatory pathways (114, 154). Siltuximab is a chimeric monoclonal antibody that also binds IL-6; however, no studies have been published on its use in the management of CRS in cancer patients to date (96).

Next generation novel immunotherapeutics could also affect COVID-19-related incidence and outcomes. For instance, AXL is a receptor tyrosine kinase which mediates tumor invasion, metastasis, and epithelial-mesenchymal transition. AXL also negatively modulates cancer immune responses through signaling pathways involving dendritic cells, natural killer cells and macrophages (155). Given its role in cancer metastasis and immune function, numerous AXL inhibitors are being used in clinical trials to treat advanced malignancies. AXL mediates viral entry into cells and modulates inflammatory responses induced by viral infections (156, 157). AXL is also overexpressed on myocardial cells in patients with heart failure and in patients who experience LV remodeling after STEMI (158). It is conceivable that through immune and cardiovascular impacts, investigational drugs that target AXL may impact outcomes of cancer patients with COVID-19 infection, and clinical trial sponsors and investigators should be encouraged to track and study COVID-19-infected trial patients to better understand these complex interactions. To this end, bemcentinib, an oral AXL inhibitor under investigation as a cancer immunotherapeutic, has recently been repurposed to combat COVID-19 as part of the Accelerating COVID-19 Research & Development (ACCORD) platform in the United Kingdom.

Interestingly, human antibodies have been isolated from the convalescent serum of COVID-19 survivors and when coupled have been shown to be protective. Perhaps the use of these emerging dual antibodies may be as efficacious for COVID-19 patients as the dual antibodies trastuzumab and pertuzumab have been for breast cancer patients. Developing therapeutics from antibodies such as these may help provide safer effective options for COVID-19 patients and facilitate avoiding potential or proven drug-drug interactions.

### Role of CYP450 and Drug Transporters

The antiviral drug remdesivir is an investigational drug being used to treat COVID-19, and concomitant use with drugs that are strong CYP3A4 inducers is not recommended (159). The CYP450 enzyme system (which includes CYP3A4) forms the backbone for metabolism of multiple drugs and plays a vital role in metabolism of numerous cardio-oncologic drugs including beta blockers, calcium channel blockers, statins, cyclophosphamide, docetaxel, cisplatin, and tyrosine kinase inhibitors (160). In particular, the antiandrogen drugs apalutamide and enzalutamide used to treat prostate cancer are strong CYP3A4 inducers (161–163). Both agents can associate with CVT, such as atrial fibrillation, hypertension, and ischemic heart disease, especially in individuals with pre-existing cardiovascular diseases (161–164). Concurrent use of remdesivir with these drugs should be avoided at the interface of COVID-19 and cardio-oncology. Of note, in cardio-oncology, the calcium channel blockers diltiazem and verapamil are moderate inhibitors of CYP3A4, which also metabolizes cancer pharmacologic drugs such as doxorubicin, imatinib, and ibrutinib (160). *In vitro*, remdesivir is a substrate for CYP2C8, CYP2D6, and CYP3A4, and an inhibitor of CYP3A4, as well as a substrate for p-glycoprotein and organic anion transporting polypeptides 1B1 (OATP1B1) and an inhibitor of OATP1B1 (165). P-glycoprotein and OATP1B1 are membrane transporters known to help mediate drug-drug interactions. However, remdesivir generally has a low potential for clinically significant drug-drug interactions mediated by the CYP450 system or drug transporters (165–167), since remdesivir functions as a prodrug that is rapidly metabolized to the active bioavailable form (165, 168).

### Anticoagulation

Empiric therapeutic anticoagulation associates with better prognosis in severe COVID-19 cases, with improved in-hospital mortality in retrospective analyses (129, 169). While practice has varied across centers during the pandemic, anticoagulation should be considered based on COVID-19 patient factors and risk stratification (119).

Empiric prophylactic anticoagulation also associates with better outcomes in cancer patients who are hospitalized and have reduced mobility, or are ambulatory and have (170, 171):

- advanced or metastatic pancreatic cancer,
- intermediate-high VTE risk based on cancer type or Khorana score,
- or treatment with immunomodulatory drugs and steroids or other systemic antineoplastic therapies.

### Neurohormonal Drugs: ACEIs and ARBs

It has been suggested that ACE inhibitors may counteract resulting unopposed ACE-mediated effects in COVID-19 (81). Thus, the influence of these vasoactive and cardiovascular remodeling drugs on the risk and severity of COVID-19 has been under investigation, with some studies suggesting benefit, juxtaposed with initial speculations about harm (28, 172–181). It is unknown whether polymorphisms in ACE, or polymorphisms in ACE2 that may contribute to COVID-19 prognosis [see pre-prints (182, 183)], also determine the prognosis of patients in cardio-oncology treated with RAAS regulators, such as ACE inhibitors and ARBs.

It is important to note that ACEIs and ARBs have established benefits in protecting the myocardium. They are among first-line therapy for various CVT (e.g., cardiomyopathy, hypertension, and myocardial infarction) in cancer patients and survivors, along with beta blockers, to mitigate symptoms and prolong survival (184). Withdrawal of these agents instigates clinical decompensation in high-risk patients, such as rapid relapse of dilated cardiomyopathy in cancer patients with CVT due to neoplastic agents (28, 172, 185). Consequently, patients receiving ACEIs or ARBs should continue ACEI/ARB therapy during the COVID-19 pandemic (172, 186, 187). Taken together, these findings suggest overlapping utility of these drugs in both cardio-oncology and COVID-19.

### Implications of Common Pharmacology

It is also important to study shared pharmacologic management opportunities in COVID-19 and cardio-oncology. Corticosteroids and immunomodulatory drugs such as tocilizumab and bemcentinib and other analogous therapies are being used or studied in cardio-oncology and have also been repurposed during the pandemic to temper the inflammation milieu initiated by SARS-CoV-2. Further, thousands of cancer patients are currently enrolled in clinical trials combining ICIs with investigational novel therapeutics across the world, accounting for another special risk population in the COVID-19 pandemic. Ongoing clinical trials on anti-interleukins in COVID-19 patients (188, 189) (NCT04330638, NCT04317092) will also help us to elucidate benefits and outcomes. Accordingly, an algorithm has been proposed to incorporate anti-inflammatory agents such as tocilizumab, canakinumab (IL-1β monoclonal antibody), anakinra, etanercept, and infliximab (TNFα monoclonal antibody) to curb CRS in acute COVID-19 infection (190). The antiviral remdesivir carries a low risk of modulating the membrane drug transporter p-glycoprotein and the cytochrome protein 450 family of enzymes and potentially interacting with CV and cardio-oncology drugs. Nevertheless, caution is recommended with the combined use of any drugs that modulate p-glycoprotein or CYP450, due to their potential for drug-drug interactions and resultant effects on the CV system in cardio-oncology and COVID-19. Therapeutic anticoagulants are another class of medications found to be useful in both COVID-19 and cardio-oncology, due to their beneficial effects on thromboembolism. Clinical studies are being pursued to determine the impact of direct oral anticoagulants or aspirin and statin to limit arterial or venous thrombotic risk in cancer (120). Regulators of the RAAS (primarily ACEI/ARB) have also taken centerstage, as many patients are on these medications to treat hypertension or other common comorbidities that increase the risk of a more severe course in those with COVID-19. There has been debate about whether these RAAS modulator drugs augment the risk of COVID-19 infection, given the virus' use of the ACE receptor to enter host cells. ACE receptor gene polymorphisms have also been implicated in the prediction of disease severity, with questionable regulation by the ACEI/ARB drug class. Pursuit of observational studies and clinical trials continues to help elucidate the impact of ACEIs and ARBs in the pandemic (27). Further study should also define the interplay between SARS-CoV-2 and RAAS and explore any differential effects of ACEI vs. ARB therapy and tumor specific responses (91, 93, 94, 185). Future collaboration among basic and clinical scientists should focus on the biological rationale for the treatment of COVID-19 patients as well as limited understanding with respect to the interaction of RAAS inhibitors, ACE2 levels and SARS CoV-2 infectivity in humans (27).

## Discussion

As we recover from the COVID-19 pandemic, we should not let opportunities for learning surreptitiously slip from our grasp. The myriad of overlap of CV toxicities, pathophysiology, and innovative management in COVID-19 and cardio-oncology provide multiple paths for exploration that could lead to greater understanding of both COVID-19 and CVT noted in cardio-oncology (Figure 1). It would behoove us in cardio-oncology to continue to closely study these toxicities, pathophysiology, and pharmacologic options in COVID-19 to help update our understanding in cardio-oncology. Cardio-oncology continues to expand as a relatively new medical subspecialty. Knowledge gaps in CVT toxicity, pathophysiology, and pharmacology in cardio-oncology may benefit from the application of novel concepts, paradigms, and drug use from overlapping forms of CVT in COVID-19 (Table 1).

In addition to short-term morbidity and mortality, patients who recover from COVID-19 infection may be at increased risk of future incident CVD and CVD-related complications (191, 192). Severe acute respiratory syndrome coronavirus (SARS-CoV-1) and Middle East respiratory syndrome coronavirus (MERS-CoV) infection have been implicated in causing diabetes, hypertension and altered lipid metabolism (193–195). The increase in CVD risk profile combined with the possibility of viral-mediated impairment in cardiac and/or pulmonary function may combine to further increase the risk and complexity of future CVD events. Aggressive risk factor modification and prophylactic therapy may prove important in mitigating long-term CVT. Optimal prevention and management of CVT will require a multidisciplinary approach with close collaborations among various medical specialties and researchers.

As our current practices change and new questions arise, future studies in cardio-oncology should focus on studying the emerging cardiovascular epidemiology of COVID-19, as well as the impact of changed practices on the health of patients with cancer and CVD. To reduce the rate of transmission while providing safe and timely care for patients with cancer and CVD, temporary recommendations favored telehealth visits in telecardio-oncology and deferral of non-urgent procedures, similar to the rest of the population (10, 196). Cardiac imaging surveillance was limited to patients who were more likely to have abnormal testing or at higher risk for cancer-related CVT, particularly if test results would guide initiation of cardioprotective medications or impact cancer therapy delivery (197).

Of utmost importance is ensuring equity in our distribution of hope, health, and healing in the midst of and beyond the pandemic, as we extend lessons learned from COVID-19 to cardio-oncology. Ethnic health disparities during the pandemic have amplified a pre-existing broken healthcare structure, with disproportionate percentages of African-Americans severely and fatally affected by COVID-19 (69–73). The pandemic has been set on a backdrop of inequity, in which African-American cancer patients are known to be more susceptible to CVT following cancer therapies (64–68). The higher risk for African Americans in both COVID-19 and cardio-oncology is of multifactorial etiology, including higher rates of CVD and CVD risk factors, which are often also underdiagnosed and undertreated (57, 69, 198–206). Underlying causes of the plethora of inequalities in healthcare are largely structural and socioeconomic and reflect our imperfections as a society, with socioeconomic status being a risk factor for CVD, CVT, and COVID-19 (198, 207, 208). We must recognize the imbalance of comorbidities and sociodemographics in ethnic populations, in order to make equitable progress in the post-pandemic era. The lasting impact of COVID-19 in cardio-oncology need not be the challenges we faced while caring for our patients during the pandemic. The long-term sequelae should be steps we have taken to optimize quality and quantity of life for all.

Thus, there are several areas of overlap, similarity, and interaction in the toxicity, pathophysiology, and pharmacology profiles in COVID-19 and cardio-oncology syndromes. Learning more about either will likely provide some level of insight into both, with further illumination of CVT mechanisms and new paradigms of drug utilization to help guide research and clinical practice in both COVID-19 and cardio-oncology. Such an approach can be informative peri-pandemic, and should perhaps be pursued long after the pandemic, to assess for evidence of long-term independent or synergistic CV effects in survivors of COVID-19 and cancer, with equity at the forefront of our efforts.

## Data Availability Statement

The original contributions generated in the study are included in the article, further inquiries can be directed to the corresponding author.

## Author Contributions

S-AB conceived, designed, and helped draft the manuscript. SZ, PM, JT, BT, DI, EW, GA, JR, JS, and DJ helped draft the manuscript. BK and MW helped design and draft the manuscript. All authors revised and approved the manuscript.

## Conflict of Interest

The authors declare that the research was conducted in the absence of any commercial or financial relationships that could be construed as a potential conflict of interest.
